# Exploring the Potential of ChatGPT-4o in Thyroid Nodule Diagnosis Using Multi-Modality Ultrasound Imaging: Dual- vs. Triple-Modality Approaches

**DOI:** 10.3390/cancers17132068

**Published:** 2025-06-20

**Authors:** Ziman Chen, Nonhlanhla Chambara, Shirley Yuk Wah Liu, Tom Chi Man Chow, Carol Man Sze Lai, Michael Tin Cheung Ying

**Affiliations:** 1 Department of Health Technology and Informatics, The Hong Kong Polytechnic University, 11 Yuk Choi Rd., Hung Hom, Kowloon, Hong Kong; 2 School of Healthcare Sciences, Cardiff University, Cardiff CF14 4XN, UK; 3 Department of Surgery, The Chinese University of Hong Kong, Prince of Wales Hospital, Shatin, New Territories, Hong Kong

**Keywords:** large language model, ChatGPT, thyroid nodule, ultrasound, medical imaging

## Abstract

**Background/Objectives** Recent advancements in large language models, such as ChatGPT-4o, have created new opportunities for analyzing complex multi-modal data, including medical images. This study aims to assess the potential of ChatGPT-4o in distinguishing between benign and malignant thyroid nodules via multi-modality ultrasound imaging: grayscale ultrasound, color Doppler ultrasound (CDUS), and shear wave elastography (SWE). **Materials and Methods** Patients who underwent thyroid nodule ultrasound examinations and had confirmed pathological diagnoses were included. ChatGPT-4o analyzed the multi-modality ultrasound data using two approaches: (1.) a dual-modality strategy which employed grayscale ultrasound and CDUS, and (2.) a triple-modality strategy which incorporated grayscale ultrasound, CDUS, and SWE. The diagnostic performance was compared against pathological findings utilizing receiver operating characteristic (ROC) curve analysis, while consistency was evaluated through *Cohen’s Kappa* analysis. **Results** A total of 106 thyroid nodules were evaluated; 65.1% were benign and 34.9% malignant. In the dual-modality approach, ChatGPT-4o achieved an area under the ROC curve (AUC) of 66.3%, moderate agreement with pathology results (*Kappa* = 0.298), a sensitivity of 70.3%, a specificity of 62.3%, and an accuracy of 65.1%. Conversely, the triple-modality approach exhibited higher specificity at 97.1% but lower sensitivity at 18.9%, with an accuracy of 69.8% and a reduced overall agreement (*Kappa* = 0.194), resulting in an AUC of 58.0%. **Conclusions** ChatGPT-4o exhibits potential, to some extent, in classifying thyroid nodules using multi-modality ultrasound imaging. However, the dual-modality approach unexpectedly outperforms the triple-modality approach. This indicates that ChatGPT-4o might encounter challenges in integrating and prioritizing different data modalities, particularly when conflicting information is present, which could impact diagnostic effectiveness.

## Simple Summary

Thyroid nodules are common, and identifying whether they are harmless or cancerous is essential for guiding appropriate treatment. Ultrasound is a widely used, non-invasive tool for evaluating these nodules, but its accuracy often depends on the experience of the operator. This study explored the potential of ChatGPT-4o, a large language model capable of processing both text and images, to assist in diagnosing thyroid nodules using ultrasound data. We compared two diagnostic strategies: one using grayscale and color Doppler ultrasound images, and another that also included tissue stiffness measurements derived from elastography. Surprisingly, the simpler two-image approach performed better than the three-input method. Adding more diagnostic information did not always improve performance—especially when the data provided conflicting signals. In such cases, ChatGPT-4o struggled to determine which diagnostic information should be prioritized. These findings highlight both the potential and the current limitations of large language models in supporting complex clinical reasoning tasks.

## 1. Introduction

The rapid advancement of artificial intelligence (AI) has driven the development of large language models (LLMs), such as ChatGPT, which demonstrate exceptional capabilities in natural language processing [1]. Trained on extensive datasets, these models excel not only in understanding and generating text but also in performing complex tasks such as reasoning, summarization, and decision-making. Recent innovations have extended their functionality to multi-modality processing, enabling LLMs to interpret and integrate both linguistic and non-linguistic data, including medical images [2,3]. ChatGPT-4o, the latest version, exemplifies these advancements by offering improved capabilities for analyzing and synthesizing both textual and visual information [4]. In healthcare, LLMs offer considerable potential beyond automating routine tasks like documentation, providing meaningful support for clinical decision-making [5]. One promising application lies in medical imaging, where LLMs have the potential to enhance diagnostic accuracy and overall performance [6,7]. Integrating LLMs with medical imaging could improve diagnostic precision and reduce the impact of practitioner-dependent variation, thereby achieving more consistent and reliable results. This potential makes LLMs especially relevant in complex diagnostic fields such as thyroid nodule evaluation, where accurate and reliable assessments are critical for guiding patient care.

Thyroid nodule diagnosis and management present increasingly relevant clinical challenges, given that treatment strategies differ significantly between benign and malignant nodules [8]. While benign nodules often require only observation or conservative management, malignant nodules typically demand surgical intervention. Therefore, accurately distinguishing between these two categories is essential to prevent over-treatment, reduce patient anxiety, and ensure optimal use of healthcare resources. Moreover, unnecessary thyroidectomy, particularly in patients with low-risk nodules, may result in postoperative scarring, psychological distress, and reduced thyroid cancer-specific quality of life, all of which have been associated with heightened levels of decision regret [9]. Ultrasound imaging is the preferred initial diagnostic tool for evaluating thyroid nodules due to its non-invasive nature, accessibility, and cost-effectiveness [10]. It provides essential morphological information, such as nodule size, shape, echogenicity, and the presence of calcifications. However, the accuracy of ultrasound-based diagnosis depends heavily on the operator’s proficiency, which can lead to varying diagnostic outcomes [11]. Studies have reported only fair-to-moderate interobserver agreement among radiologists or endocrinologists when applying ultrasound risk stratification systems, particularly in the evaluation of subtle sonographic features [12,13]. These limitations underscore the need for standardized, objective tools that can improve diagnostic performance and ensure reliable results across clinical settings.

Compared to traditional image-specific deep learning models—such as convolutional neural networks (CNNs) trained solely on medical image datasets—LLMs like ChatGPT-4o are general-purpose systems capable of handling diverse data formats through text-based interactions, without requiring task-specific retraining [14]. This architecture enables a more flexible integration of different diagnostic inputs within a unified framework, guided entirely by natural language prompts. Additionally, LLMs offer the advantage of generating human-readable reasoning, which may enhance interpretability and transparency in diagnostic processes [15]. From a deployment perspective, LLMs are often easier to implement in clinical or low-code settings, as they bypass the need for dedicated model training pipelines or high-performance computing resources. These features position LLMs as a promising complementary tool in AI-assisted ultrasound interpretation.

Recognizing this need, recent studies have explored the integration of LLMs with medical imaging data to support clinical decision-making [16]. One emerging research focus involves LLM-based approaches for thyroid nodule diagnosis, which show promise for enhancing diagnostic accuracy [17]. In earlier work, our team investigated the feasibility of using ChatGPT-4o to classify thyroid nodules based on grayscale ultrasound images [18]. Although these efforts produced promising initial results, they also revealed significant limitation—particularly due to the reliance on a single imaging modality. This experience underscores the need for a multi-modal approach, given that physicians often combine multiple imaging techniques for more accurate diagnosis. For example, color Doppler ultrasound (CDUS) provides information about hemodynamics, helping in the identification of abnormal vascular patterns associated with malignancy [19], while shear wave elastography (SWE) measures tissue stiffness, a key parameter for distinguishing between benign and malignant nodules [20]. The integration of these complementary ultrasound imaging modalities can offer a more comprehensive understanding of nodule characteristics, reducing diagnostic uncertainty and improving clinical accuracy.

This study aims to address the limitations of previous research by integrating multiple imaging modalities—grayscale ultrasound, CDUS, and SWE—into a unified LLM-based diagnostic framework. Specifically, we explore whether this multi-modality approach can provide better diagnostic insights and enhance the performance of LLM-based diagnostic schemes in distinguishing between benign and malignant thyroid nodules.

## 2. Materials and Methods

### 2.1. Ethical Approval and Study Population

This cross-sectional study was conducted in accordance with the Declaration of Helsinki and received approval from the institutional ethics committee. All participants provided written informed consent prior to enrollment. From May 2019 to August 2021, patients scheduled for thyroid ultrasound examinations, followed by fine-needle aspiration cytology (FNAC) or thyroid surgery, were prospectively and consecutively recruited. This consecutive enrollment approach helped minimize selection bias and enhanced the representativeness of the study cohort. Inclusion criteria included patients undergoing thyroid nodule ultrasound evaluations with subsequent FNAC or thyroidectomy, which comprised complete grayscale ultrasound, CDUS, and SWE examinations. Exclusion criteria were poor-quality ultrasound images that could hinder diagnostic interpretation and inconclusive pathological diagnoses from FNAC or histopathology. Pathological diagnoses were confirmed through FNAC or histopathological examination of surgically excised thyroid nodules.

### 2.2. Ultrasound Imaging Procedure

All ultrasound examinations were conducted by a radiographer with over three years of clinical experience, utilizing the Aixplorer Ultrasound imaging system (SuperSonic Imagine, Aix-en-Provence, France) equipped with an SL15-4 linear array probe (4–15 MHz). Each examination included grayscale ultrasound, CDUS, and SWE. Grayscale ultrasound was employed to capture the largest transverse cross-sectional view of each thyroid nodule, providing structural information for evaluation. These images were saved for further analysis. Subsequently, CDUS was used to assess the vascularization patterns of the nodule, capturing blood flow within and around it, with the images stored for later review. For SWE, the stiffness of the nodule was measured by recording the mean elastic value, expressed in kPa as Young’s modulus. Three consecutive measurements were taken from the same region of the nodule, and their arithmetic mean was calculated and recorded as the final stiffness measurement. All imaging data, including grayscale ultrasound images, CDUS images, and SWE measurements, were digitally stored and de-identified to ensure patient confidentiality.

### 2.3. Application of ChatGPT-4o for Multi-Modality Analysis

**Figure 1:**
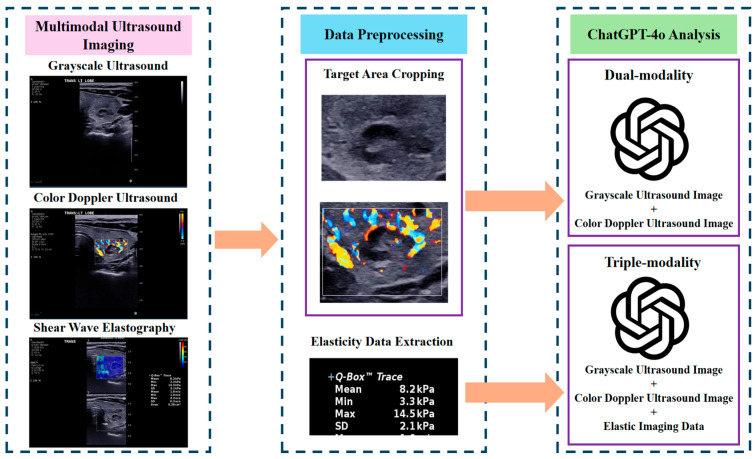
The workflow of ChatGPT-4o’s analysis for thyroid nodule classification, comparing the dual-modality approach (grayscale ultrasound image and color Doppler ultrasound image) with the triple-modality approach (grayscale ultrasound image, color Doppler ultrasound image, and shear wave elastography measurement).

This study utilized ChatGPT-4o (OpenAI, San Francisco, CA, USA), an LLM with multi-modal capabilities, to analyze the ultrasound imaging data of thyroid nodules. ChatGPT-4o does not currently support user-defined random seed settings or deterministic output modes through its public web interface. Therefore, strict output determinism could not be enforced. To ensure reproducibility, all prompts were standardized, image sequences were fixed, and SWE data were formatted uniformly across cases. The analysis workflow involved the following key stages (Figure 1):

Data Preprocessing: Grayscale ultrasound and CDUS images were preprocessed by cropping to retain only the nodule and surrounding thyroid tissue, excluding irrelevant structures like the trachea and carotid arteries. This ensured non-diagnostic areas were removed, enabling a more focused evaluation of the nodule’s characteristics.

Image and Data Upload: After preprocessing, grayscale and CDUS images were manually uploaded to the ChatGPT-4o web interface using the standard OpenAI platform. SWE measurements were incorporated into the prompt as structured text rather than as image data. All interactions with the model—including image uploads and prompt submissions—were performed manually by the research team in a controlled and standardized manner to ensure consistency across all cases. No external application programming interface (API) or plugin integration was used throughout the process. According to OpenAI’s documentation, the platform automatically resizes input images before analysis to meet internal processing constraints, typically scaling the shortest side to 768 pixels while preserving aspect ratio. Although this resizing is system-controlled and not user-configurable, prior manual preprocessing ensured that each image was centered on diagnostically relevant content. No additional compression or downscaling was applied by the research team, thereby minimizing the potential loss of diagnostic detail within the model’s technical limitations.

**Figure 2:**
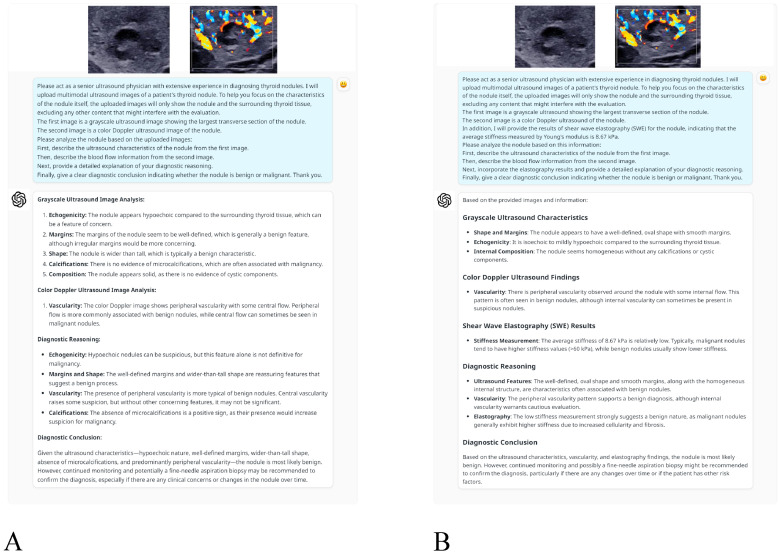
An example of ChatGPT-4o interaction for classifying thyroid nodules: (**A**) presents a dual-modality analysis using grayscale and color Doppler ultrasound images, while (**B**) incorporates shear wave elastography for a triple-modality evaluation.

Diagnostic Approaches: Two diagnostic approaches were adopted to evaluate the thyroid nodules, each guided by tailored prompts (Figure 2):

1. *Dual-modality analysis*: This approach used grayscale ultrasound and CDUS images only. The following prompt was provided to ChatGPT-4o to direct its analysis: “*Please act as a senior ultrasound physician with extensive experience in diagnosing thyroid nodules. I will upload multimodal ultrasound images of a patient’s thyroid nodule. To help you focus on the characteristics of the nodule itself, the uploaded images will only show the nodule and the surrounding thyroid tissue, excluding any other content that might interfere with the evaluation. The first image is a grayscale ultrasound image showing the largest transverse section of the nodule. The second image is a color Doppler ultrasound image of the nodule. Please analyze the nodule based on the uploaded images: First, describe the ultrasound characteristics of the nodule from the first image. Then, describe the blood flow information from the second image. Next, provide a detailed explanation of your diagnostic reasoning. Finally, give a clear diagnostic conclusion indicating whether the nodule is benign or malignant. Thank you.*”
2. *Triple-modality analysis*: This analysis used SWE measurements, grayscale ultrasound and CDUS images to offer an integrated evaluation approach. The prompt was adapted as follows: “*Please act as a senior ultrasound physician with extensive experience in diagnosing thyroid nodules. I will upload multimodal ultrasound images of a patient’s thyroid nodule. To help you focus on the characteristics of the nodule itself, the uploaded images will only show the nodule and the surrounding thyroid tissue, excluding any content that might interfere with the evaluation. The first image is a grayscale ultrasound showing the largest transverse section of the nodule. The second image is a color Doppler ultrasound of the nodule. In addition, I will provide the results of shear wave elastography (SWE) for the nodule, indicating that the average stiffness measured by Young’s modulus is XX kPa. Please analyze the nodule based on this information: First, describe the ultrasound characteristics of the nodule from the first image. Then, describe the blood flow information from the second image. Next, incorporate the elastography results and provide a detailed explanation of your diagnostic reasoning. Finally, give a clear diagnostic conclusion indicating whether the nodule is benign or malignant. Thank you.*”

Diagnostic Output: ChatGPT-4o analyzed the uploaded multi-modal ultrasound data according to the provided prompts and returned diagnostic conclusions regarding the benign or malignant nature of the nodules. The model’s reasoning incorporated the nodule’s structural features from the grayscale ultrasound image, vascular patterns from the CDUS image, and/or stiffness information derived from SWE measurements.

### 2.4. Statistical Analysis

Statistical analyses were conducted using SPSS 26.0 and R version 4.2.0. Continuous variables were presented as mean ± standard deviation or median (interquartile range [IQR]), and categorical data as frequencies (percentages). Pathological findings were used as the gold standard for evaluating the model’s diagnostic performance. *Cohen’s Kappa* value assessed the agreement between ChatGPT-4o’s diagnostic results and the gold standard, with interpretation guidelines as follows: 0–0.2 (poor), 0.2–0.4 (fair), 0.4–0.6 (moderate), 0.6–0.8 (substantial), and 0.8–1.0 (almost perfect). Receiver operating characteristic (ROC) curve analysis was used to evaluate diagnostic performance, and the area under the ROC curve (AUC) was calculated. Sensitivity, specificity, accuracy, positive predictive value (PPV), and negative predictive value (NPV) were reported for both approaches. A post hoc power analysis was conducted to evaluate whether the current sample size was adequate for detecting a meaningful level of agreement. Assuming a true *Kappa* value of 0.30 and a null hypothesis of κ = 0 (i.e., no agreement beyond chance), the study with 106 subjects and category frequencies of 0.35 and 0.65 achieved approximately 87% power. A two-sided *p*-value < 0.05 indicated significance.

## 3. Results

### 3.1. Study Population Characteristics

The study included 102 patients (84 females, 18 males) with a mean age of 53.71 ± 12.46 years. A total of 106 thyroid nodules were evaluated, of which 69 (65.1%) were classified as benign and 37 (34.9%) as malignant based on pathological findings. The median elasticity for benign nodules was 14.30 kPa (IQR: 9.73–18.27), while malignant nodules demonstrated a higher median value of 17.47 kPa (IQR: 12.00–30.74) (Table 1).

### 3.2. Concordance with Pathological Diagnosis

**Figure 3:**
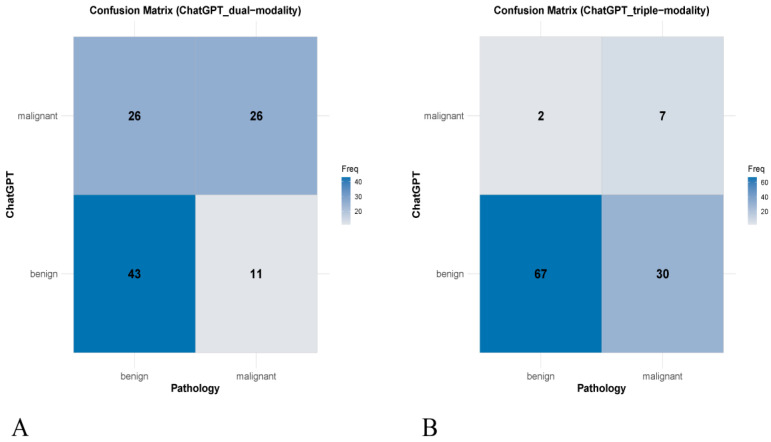
Confusion matrices for thyroid nodule classification using ChatGPT-4o: (**A**) dual-modality approach and (**B**) triple-modality approach.

The dual-modality approach showed moderate agreement with pathology (*Kappa* = 0.298, 95% confidence interval [CI]: 0.123–0.473, *p* = 0.001). This method accurately classified 43 benign and 26 malignant nodules but misclassified 26 benign nodules as malignant and 11 malignant nodules as benign. In contrast, the triple-modality approach demonstrated lower agreement (*Kappa* = 0.194, 95% CI: 0.038–0.350, *p* = 0.014), correctly identifying 67 benign nodules, but misclassifying 30 malignant nodules as benign and 2 benign nodules as malignant (Table 2). The confusion matrices for both approaches are illustrated in Figure 3.

### 3.3. Diagnostic Performance Comparison

**Figure 4:**
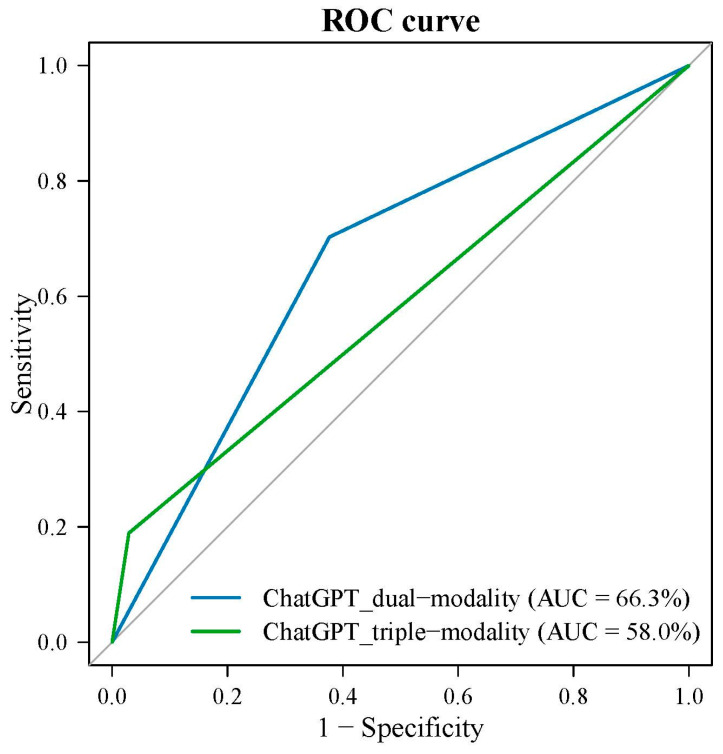
ROC curves comparing the performance of ChatGPT-4o in thyroid nodule classification using dual-modality and triple-modality approaches.

The dual-modality approach demonstrated a sensitivity of 70.3% (95% CI: 53.0–84.1) and a specificity of 62.3% (95% CI: 49.8–73.7), resulting in an overall accuracy of 65.1% (95% CI: 55.2–74.1). The PPV and NPV were 50.0% (95% CI: 40.9–59.1) and 79.6% (95% CI: 69.7–86.9), respectively, with an AUC of 66.3% (95% CI: 56.5–75.2). The triple-modality approach demonstrated a higher specificity of 97.1% (95% CI: 89.9–99.6). However, this was accompanied by a substantially lower sensitivity of 18.9% (95% CI: 8.0–35.2), leading to an overall accuracy of 69.8% (95% CI: 60.1–78.4). The PPV and NPV for the triple-modality model were 77.8% (95% CI: 43.4–94.1) and 69.1% (95% CI: 65.5–72.4), respectively. However, the AUC for the triple-modality approach was lower, at 58.0% (95% CI: 48.0–67.5) (Table 3). The ROC curves comparing the two approaches are presented in Figure 4.

### 3.4. Representative Misclassified Case

To enhance interpretability, we present a representative case in which the dual-modality approach correctly identified a malignant nodule, whereas the triple-modality approach misclassified it. The patient was a 61-year-old man with a thyroid nodule confirmed as malignant by histopathology.

**Figure 5:**
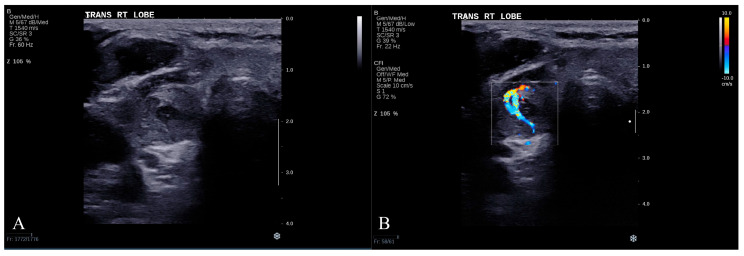
A representative misclassified case. (**A**) Grayscale and (**B**) color Doppler ultrasound images of a malignant thyroid nodule. The images are shown uncropped to preserve the full anatomical context for the reader.

ChatGPT-4o’s analysis of the grayscale ultrasound image (Figure 5A) described the nodule as solid and markedly hypoechoic, with irregular margins and a taller-than-wide shape—features interpreted as highly suggestive of malignancy. Its analysis of the CDUS image (Figure 5B) revealed rich internal vascularity with a penetrating, centripetal pattern, further reinforcing the malignant impression. Based on this dual-modality input, ChatGPT-4o correctly classified the nodule as malignant.

In a subsequent independent session using triple-modality data—including grayscale, CDUS, and a SWE value of 11.87 kPa—the model revised its diagnosis and classified the lesion as benign. This low stiffness measurement, within the typical benign range, appeared to outweigh the suspicious grayscale and Doppler features. This misclassification illustrates a potential limitation of ChatGPT-4o in reconciling conflicting information across modalities.

## 4. Discussion

In this study, we explored the diagnostic capabilities of ChatGPT-4o for thyroid nodules by analyzing multi-modality ultrasound imaging, specifically integrating grayscale ultrasound, CDUS, and SWE. Two analytical frameworks were employed: a dual-modality approach (combining grayscale ultrasound and CDUS images) and a triple-modality approach (which further includes SWE measurements). Our findings reveal significant variability in ChatGPT-4o’s performance across these approaches, with the dual-modality strategy unexpectedly yielding superior results. This suggests potential challenges for ChatGPT-4o in effectively integrating data from multiple modalities.

Current research investigating the potential of ChatGPT-4 in medical imaging applications, particularly for thyroid nodule classification, is still in its early stages. For instance, Sultan et al. demonstrated that ChatGPT-4 is capable of identifying thyroid nodules in grayscale ultrasound images and accurately describing their features, which could aid in differential diagnoses [17]. However, this exploratory study was primarily conceptual, analyzing only a single case, which raises concerns about the generalizability of its findings to broader clinical contexts. In contrast, another study evaluated ChatGPT-4 for thyroid nodule classification using a larger sample of 116 cases [18]. Despite the increased sample size, the model exhibited limited diagnostic performance, with a *Kappa* value of 0.116 and an AUC of 57.0%, suggesting that its diagnostic capabilities are still underdeveloped. These findings underscore the need for further investigation into whether multi-modal data analysis could enhance the model’s ability to comprehensively assess lesion characteristics in clinical practice. Building on the previous studies, in our research we utilized three modalities of ultrasound imaging and data to further investigate the diagnostic potential of ChatGPT-4o. Grayscale ultrasound primarily provided structural information, CDUS assessed hemodynamics—such as intranodular and perinodular blood flow—and SWE measured tissue stiffness. In this study, we employed grayscale ultrasound images and CDUS images as inputs to ChatGPT-4o to construct a dual-modality diagnostic strategy. Furthermore, we incorporated SWE data to develop a triple-modal diagnostic strategy. The dual-modality analysis achieved an AUC of 66.3% and a *Kappa* value of 0.298, reflecting a moderate improvement over single-modality analysis reported in previous research [18]. This highlights the value of incorporating additional modalities to provide complementary diagnostic information, thereby enhancing the model’s overall performance and suggesting the potential clinical utility of dual-modality approaches. In contrast, the triple-modality analysis achieved high specificity (97.1%) but low sensitivity (18.9%), resulting in an AUC of only 58.0%. The performance of the triple-modality strategy was inferior to that of the dual-modality strategy. These findings indicate that simply inputting more ultrasound modalities into ChatGPT-4o does not necessarily lead to improved performance.

The reduced performance of the triple-modality approach may be attributed to challenges encountered during the integration of multi-modal data. Specifically, ChatGPT-4o may struggle to reconcile conflicting diagnostic information across modalities, which can constrain its overall diagnostic effectiveness. While SWE is valuable for assessing tissue stiffness and distinguishing benign from malignant lesions, its measurements can sometimes conflict with the findings from grayscale ultrasound and CDUS. Research has shown that SWE encounters difficulties in differentiating malignant thyroid nodules with low stiffness values, as their elasticity indices can substantially overlap with those of benign nodules, complicating analysis by ChatGPT-4o [21]. For instance, a malignant nodule may exhibit increased central or intranodular vascularity on CDUS—features typically associated with malignancy—while simultaneously showing a low stiffness value on SWE, which is more commonly observed in benign nodules. These conflicts can challenge the model’s decision-making, potentially leading to misclassification. To simulate real-world clinical conditions, our prompts did not explicitly direct the model to prioritize any specific modality. This design was intentional, aiming to evaluate ChatGPT-4o’s intrinsic ability to weigh and reconcile multi-modal information—mirroring how human clinicians exercise judgment based on experience rather than preset rules. Therefore, the reduced diagnostic performance in the triple-modality setting may stem not from the external prompt design, but from the model’s inherent inability to assign appropriate weight to conflicting inputs. Although ChatGPT-4o is capable of processing vast amounts of data, it lacks the refined clinical judgment and intuition that experienced clinicians possess [22,23]. Clinicians can selectively focus on relevant diagnostic information, prioritize critical data, and integrate their clinical experiences to resolve conflicting findings [24]. In contrast, ChatGPT-4o lacks the ability to distinguish between more and less informative features in conflicting inputs, which may obscure diagnostic clarity and increase uncertainty [25]. These findings suggest that the current LLM design has limitations in addressing conflicts inherent in multi-modal data processing. While ChatGPT-4o shows promise, significant challenges remain in its ability to prioritize information and integrate multi-modal data effectively. Future research should focus on developing more advanced algorithms to improve consistency and accuracy in multi-modal analysis, thereby enhancing LLM’s clinical applicability.

This study highlights the potential of ChatGPT-4o in analyzing multi-modal ultrasound imaging; however, several limitations must be acknowledged. First, as an exploratory study, the relatively small sample size may limit the generalizability of the findings. Although the agreement analysis using *Cohen’s Kappa* demonstrated statistically significant concordance between model diagnostics and pathological outcomes (*p* < 0.05), no statistical comparison of AUCs between the dual- and triple-modality models was conducted, as the current sample size was deemed insufficient to support a reliable assessment of AUC differences. Future studies with larger cohorts are warranted to validate and extend these preliminary findings. Second, although all ultrasound examinations were performed by a radiographer with three years of experience, the study did not evaluate inter- and intra-operator variability. Given that ultrasound—particularly SWE—is highly operator-dependent, the lack of variability assessment limits the generalizability and robustness of the findings. Future research should incorporate multi-operator validation to better assess reproducibility. Additionally, the current research did not evaluate ChatGPT-4o’s performance in more complex clinical scenarios, such as its ability to integrate other clinical data (e.g., laboratory test results). Future studies should explore the model’s applicability across various clinical contexts to ensure its reliability and safety in medical environments.

## 5. Conclusions

ChatGPT-4o demonstrates preliminary potential for classifying thyroid nodules using multi-modality ultrasound imaging, particularly through a dual-modality approach that combines grayscale ultrasound and CDUS images. Building on this potential, the method may serve as a supportive tool in clinical practice by assisting clinicians in distinguishing between benign and malignant nodules. However, the challenges associated with incorporating additional modalities reflect the current limitations of LLMs in effectively integrating conflicting diagnostic information. These findings underscore the need for further refinement to enhance ChatGPT-4o’s clinical applicability, warranting larger-scale validation and algorithmic advancement to improve reliability and consistency.

## Figures and Tables

**Table 1:** Baseline characteristics of study dataset.

Characteristic	Total	Benign	Malignant
Patients	102	69	33
Sex (Male/Female)	18/84	11/58	7/26
Age (years)	53.71 ± 12.46	53.23 ± 12.22	54.70 ± 13.08
Nodules	106	69 (65.1)	37 (34.9)
Nodule elastic value (kPa)	14.64 (10.30–21.24)	14.30 (9.73–18.27)	17.47 (12.00–30.74)

Notes: Categorical variables are presented as n (%) and continuous variables as mean ± standard deviation or median (interquartile range), as appropriate.

**Table 2:** Consistency analysis between diagnostic approaches and pathological results.

Index		Pathological Result		*Cohen’s Kappa* Value (95% CI)	*p* Value
		Benign	Malignant		
Dual-modality	Benign	43	11	0.298 (0.123–0.473)	0.001
	Malignant	26	26		
Triple-modality	Benign	67	30	0.194 (0.038–0.350)	0.014
	Malignant	2	7		

**Table 3:** Diagnostic performance of various diagnostic approaches.

Index	Sensitivity% (95% CI)	Specificity% (95% CI)	Accuracy% (95% CI)	PPV% (95% CI)	NPV% (95% CI)	AUC% (95% CI)
Dual-modality	70.3 (53.0–84.1)	62.3 (49.8–73.7)	65.1 (55.2–74.1)	50.0 (40.9–59.1)	79.6 (69.7–86.9)	66.3 (56.5–75.2)
Triple-modality	18.9 (8.0–35.2)	97.1 (89.9–99.6)	69.8 (60.1–78.4)	77.8 (43.4–94.1)	69.1 (65.5–72.4)	58.0 (48.0–67.5)

Abbreviations: PPV, positive predictive value; NPV, negative predictive value; AUC, area under the curve; CI: confidence interval.

## Data Availability

The data presented in this study are available from the corresponding author upon reasonable request. Data is not publicly available due to privacy or ethical concerns.
